# Should we ‘hug a hoodie’? Protocol for a systematic review and meta-analysis of interventions with young people not in employment, education or training (so-called NEETs)

**DOI:** 10.1186/2046-4053-3-73

**Published:** 2014-07-07

**Authors:** Emily J Oliver, Lauren Mawn, Helen J Stain, Clare L Bambra, Carole Torgerson, Anita Oliver, Chris Bridle

**Affiliations:** 1 School of Applied Social Sciences, Durham University, Durham DH1 3HN, UK; 2 School of Medicine, Pharmacy and Health, Durham University, Durham TS17 6BH, UK; 3 Department of Geography, Durham University, Durham TS17 6BH, UK; 4 School of Education, Durham University, Durham DH1 3LE, UK; 5 Queens Crescent Community Association, Camden, London NW5 4QE, UK; 6 School of Sport and Exercise Sciences, Aberystwyth University, Aberystwyth SY23 3FD, UK

**Keywords:** Unemployment, Effectiveness, Education, Engagement, Well-being

## Abstract

**Background:**

Whilst the majority of young people succeed in education and make a positive transition to the world of work and adult life, recent statistics identify that youth comprise 40% of the world's unemployed, equating to nearly 75 million individuals. These numbers are associated with both decreased economic activity and adverse well-being, with accompanying social, health and financial costs. As a result, a wide range of providers have implemented interventions targeting this population; however, their relative effectiveness is unknown. This is exacerbated by a diverse literature base, the delivery of provision and policy across multiple sectors and disparate approaches to programme evaluation.

**Methods and design:**

We will undertake a systematic review of interventions targeting youth not in employment, education or training (NEET) populations. Only randomised and non-randomised controlled trials will be included. The objectives of the review will be to: (i) systematically review, synthesise and quality appraise experimental evidence on the effects of interventions with NEET young people, (ii) estimate effects on current NEET status, well-being and other relevant psychological and behavioural outcomes, (iii) investigate potential variation in intervention effects among sub-groups stratified by pre-trial duration of current status, socioeconomic status, gender, sub-classifications of NEET individuals and intervention components (e.g. type, frequency, duration, provider and setting) and (iv) assess the robustness of results in separate sensitivity analyses that exclude studies with higher risk of bias (e.g. in terms of study quality) or follow-up length. A rigorous literature search of English language publications post-1990 will be conducted using the following electronic databases: Medline, Embase, PsycINFO, ERIC, EPPI-Centre (Bibliomap), Social Science Citation Index, British Education Index, Conference Proceedings Index, Dissertation Abstracts, Popline and grey literature collections (e.g. GLADNET). These database searches will be supplemented with hand searching, requests for unpublished literature and website searches.

**Discussion:**

A report and executive summary will be developed by the research team with input from consultant stakeholders to aid translation of the findings into practice. The research will be disseminated at national and international conferences and submitted for peer-reviewed publication.

**Systematic review registration:**

PROSPERO CRD42014007535

## Background

Adolescence is a period of rapid physical, emotional and social growth. Young people are faced with significant developmental challenges including the establishment of a stable identity, mastery of personal relationships and the achievement of major educational and vocational goals. Many young people lack the socio-emotional skills necessary to successfully negotiate the transition through adolescence and are at increased risk of disengaging from family and community. Whilst the majority of young people succeed in education and make a positive transition to adult life and the world of work, a challenge exists in terms of opportunities for young people. Global youth unemployment has reached 13.1%, almost three times as high as the adult unemployment rate [1], equating to nearly 75 million individuals. This is a widespread concern, with rates of youth not in employment, education or training (NEET) reported at 21.4% in the European Union, 17.4% in North America, 26.5% in the Middle East and 27.9% in Africa [2]. In the United Kingdom (UK), recent statistics identify that despite claims of an economic recovery, 1.04 million 16–24-year-olds were not in education, employment or training [3]. For every single one of these individuals, commonly referred to in policy discourse as NEETs, the average lifetime direct cost to the public sector is £56,500 and the wider resource cost to the economy, including lost output, is estimated at £104,300 [4]. As a population, this has been projected to cost the UK up to £77 billion per annum. Further, global youth unemployment has increased by 3.4 million since 2007 [5] and rates of NEET individuals and those in vulnerable employment continue to rise [1].

The NEET population is comprised of individuals in a variety of situations requiring wide-ranging levels and types of support. For example, the UK's NEET population is comprised of 150,000 16–17-year-olds who may need additional opportunities or support to re-engage in education or training, 523,000 18–24-year-olds who are unemployed, not in education and looking for work, 249,000 who have been unemployed for over 6 months and may need significant help to find work and 490,000 18–24-year-olds who are economically inactive [6]. Moreover, this is an increasing problem with data showing that the number of young people out of work for longer than 2 years increased by 188% from 2008 to 2013 [7].

Once disengaged, youth are at risk of a range of adverse outcomes, such as reduced social and community participation in young adulthood and beyond. Further to decreased economic activity, unemployment has well-established negative consequences in terms of adverse well-being and health [8-11] as well as negative attitudes towards the self [12]. This association extends to clinical disorders, with this population presenting a greater risk of psychiatric disorders, substance use and suicidal behaviour [13]. These risks may contribute to the claims made that there is a mortality rate of 15% of long-term NEETs at 10-year follow-up, that is, almost a one in seven chance of dying [14]. This has been compared to a 1 in 358 chance of dying within 10 years if diagnosed with HIV as a 16–24-year-old and a 1 in 50 chance of dying within 10 years of a testicular cancer diagnosis [15].

Disengagement from education and labour may increase the risk of psychopathology either by failing to provide structure and the necessary developmental experiences or by increasing exposure to other disenfranchised or non-normative peers [16-18]. However, it is also apparent that mental health may facilitate a successful transition from school to work, given the association of poor mental health with a disengaged status above and beyond social disadvantage [13]. Thus, whilst disengagement is a risk factor for mental health impairments, pre-existing difficulties will also contribute to an increased likelihood of disengagement. Exploring the potency of interventions targeting not only status changes but also psychological well-being and attitudes likely to protect against negative outcomes is important for identifying effective strategies with this population.

A range of different approaches to interventions are evident in the literature, including information, advice and guidance [19], one-to-one support and esteem-building [20], informal learning programmes [21] and volunteering programmes and working life familiarisation [22]. In addition, reports have argued that some NEET individuals may have complex needs requiring a targeted psychological approach including counselling, mentoring and motivational interviewing [22]. Whilst trials focus on a multitude of outcomes for young people, an overview in terms of evidence for the relative effectiveness of these intervention approaches is not currently available. Further, there is limited understanding of the differential effectiveness of interventions dependent on the broader cultural and economic context in which they are delivered. For example, similar interventions may work well in urban but not rural areas, during economic growth but not decline or in conservative but not liberal societies. This information will be used to interpret divergent findings and subsequent recommendations.

Given the difficulties of a diverse research base contributed to by scientific, statutory and voluntary organisations, a systematic review that collates and critiques available evidence within this area will be a timely and important contribution to research. The review will include any intervention working with a population of young people who are not in education, employment or training. Outcomes of primary interest will include changes in objective status (i.e. a transition from NEET to non-NEET status), as well as key indicators of psychological well-being. This will include quality of life, recognised as physical, psychological and social functioning, as well as more specific self-directed attitudes linked to both well-being and future engagement (i.e. self-esteem and confidence). Trials will not be excluded based on outcome as the review aims to provide a comprehensive overview of experimental trials. Hence, additional outcomes may include indicators of mental illness (e.g. depression, anxiety), financial status and behaviours (e.g. offending, antisocial behaviour).

The overarching aim of this systematic review is to identify, synthesise and evaluate evidence of the effects of interventions targeting the social re-engagement and increased well-being of youth classified as not in education, employment or training. Specifically, we will: (i) systematically review, synthesise and quality appraise experimental evidence on the effects of interventions with young people classified as NEET, (ii) estimate effects on current NEET status, well-being and other relevant psychological and behavioural outcomes, (iii) investigate potential variation in intervention effects among stratified sub-groups (e.g. pre-trial duration of current status, socioeconomic status, gender), sub-classifications of NEET individuals (e.g. young offenders, teenage parent, young people with special educational needs, young carers, care leavers etc.) and intervention components (e.g. type, frequency, duration, provider and setting, cultural context of study) and (iv) assess the robustness of results in separate sensitivity analyses that exclude studies with higher risk of bias (e.g. in terms of study quality) or follow-up length.

## Methods

### Search strategy

#### Study identification

A standardised search strategy with defined terms (see Table 1) will be used to search English language papers from 1990 to present in each database search. We justify narrowing our focus given that, first, the vast majority of scientific articles are published in English and comprehension of sources would potentially be compromised by translation. Second, we suggest that target interventions are best understood in a contemporary context, hence use of the conventional inclusion threshold above. It should be noted that this is also consistent with previous topical reviews [23]. Key studies conducted prior to 1990 identified through hand-searching and reference lists will be included. We will search the following databases as indices of psychological, educational, social and health-based studies, including collections of grey literature: Medline, Embase, PsycINFO, ERIC, EPPI-Centre (Bibliomap), Social Science Citation Index, British Education Index, Conference Proceedings Index, Dissertation Abstracts, Popline and grey literature collections (e.g. GLADNET). We will supplement database searching with internet searching (e.g. Google Scholar), forward and backward citation tracking from systematic reviews and included studies and contact with study authors and research groups. A search for completed but not yet published trials will also be conducted using metaRegister at Current Controlled Trials, and authors of any such trials will be contacted for details about prospective publication dates. In addition, aid organisations with an interest in the target population will be approached for internal reports (e.g. Prince's Trust, Springboard, Tomorrow's People, Barnardo's).

**Table 1:** Sample search strategy

	**Sample search strategy**
1	Adolescen*
2	Child*
3	Teen*
4	Youth*
5	Young*
6	Student*
7	Minors
8	Juvenile
24	1 OR 2 OR 3 OR 4 OR 5 OR 6 OR 7 OR 8
9	NEET
10	Unemploy*
11	‘Out of work’
12	‘Socially excluded’
13	Disadvantaged
14	‘Not in work’
15	‘Not in Employment’
16	‘Not in education’
25	9 OR 10 OR 11 OR 12 OR 13 OR 14 OR 15 OR 16
17	‘Randomised controlled trial’
18	‘Randomized controlled trial’
19	‘Cluster trial’
20	‘Clinical trial’
21	‘Control* trial’
22	‘Control group’
23	‘Experimental group’
26	17 OR 18 OR 19 OR 20 OR 21 OR 22 OR 23
27	24 AND 25
28	24 AND 26
29	25 AND 26
30	24 AND 25 AND 26

#### Study selection

Search results will be downloaded into Endnote. Following the removal of duplicate citations, titles and abstracts will be screened independently by two reviewers against the inclusion criteria. For studies that cannot be excluded in the initial screen, full text papers will be obtained and assessed for relevance against inclusion criteria again by two reviewers independently, with discrepancies resolved through discussion or, if necessary, by recourse to a third reviewer. Search results, screening outcomes and selection decisions will be documented and presented in flow diagram format.

### Eligibility criteria

#### Studies

The review will include all studies that evaluate the effect of an intervention among NEET individuals. To identify an effect, the included studies will use experimental designs such as randomised and quasi-randomised controlled trials. Pre/post, cross-sectional and non-comparison group designs will not be included as studies of these designs provide limited evidence of effectiveness.

#### Population

The populations of interest will be young people aged between 16 and 24 who were not in employment or education (or training) at the time of the intervention commencing. We will include studies for which the mean sample age falls between 16 and 24 years. If data combines NEET and non-NEET populations, and effects for NEET cannot be separately estimated, that sample will be excluded; however, authors will be contacted to attempt to obtain unpublished separate analyses where these are available.

#### Intervention

All interventions delivered to the target population will be included, such as community-based interventions and outreach, psychological and counselling support, media campaigns, career development initiatives and educational programmes. Studies of multi-component interventions will be included.

#### Comparison

Any study with a concurrent control or comparison group (including usual treatment controls) will be included.

#### Outcomes

The review will include studies that report data from a valid assessment of NEET status, psychological or behavioural variables, including (but not limited to) current employment and financial status, quality of life and physical and mental well-being, confidence, efficacy and esteem. We define quality of life as the extent to which health affects individuals' lives, well-being relates to physical, psychological and/or social functioning. For these concepts, we will include existing measures of well-being, quality of life or their components validated with general populations [24-27] as well as population-specific measures [28].

Psychological variables will include subjective measures of confidence in or satisfaction with oneself and one's ability to function in society. Examining the impact of interventions on subjective self-report measures of confidence in or satisfaction with the self is important given the predictive potency of these variables for cognitions, choices and goal-directed behaviour [29]. Global- and population-specific measures of self-confidence, relevant self-efficacy measures (e.g. perceived empathic and social efficacy scale) and measures of self-esteem [30,31] will be included.

Lastly, assessments of NEET status will include those in which individuals are classified as engaged (if they are in education employment or training) or not engaged (if not in employment, education or training). Quality of employment will be noted if recorded (e.g. underemployment, International Standard Classification of Occupations groupings) [32]. Other objective measures of NEET status will be included if transparent and relevant (e.g. credit risk); hence, additional outcomes reported are expected to emerge from the literature.

### Data extraction and quality appraisal

We will develop, test and refine a structured data extraction template. Data will be extracted from each study by each reviewer, and these will be cross-checked for accuracy. First or corresponding authors will be contacted for additional information where necessary. The template will encompass methodological characteristics (e.g. design, unit of randomisation, length of follow-up) and sample characteristics (e.g. prior length of NEET status), description of the intervention and control conditions (e.g. content, level, structure, theoretical basis, type, frequency, duration, provider and setting, cultural context of study), measures and outcomes for baseline and all follow-up periods and process-related outcomes (e.g. recruitment approach, uptake and dropout).

Using the Cochrane risk of bias assessment tool, two reviewers will independently assess each study for the adequacy of key quality issues in the individual studies, for example, method of randomisation (sequence generation, allocation concealment, independence), blinding and completeness of follow-up. Each domain will be assessed as adequate, unclear or inadequate, according to established criteria. The overall risk of bias for each study will be classified and interpreted as follows: (a) low risk of bias, all criteria graded adequate, that is, plausible bias unlikely to seriously alter results, (b) unclear risk of bias, one criterion not adequate, that is, plausible bias that raises some doubt about results and (c) high risk, greater than one criterion inadequate, that is, plausible bias that seriously weakens confidence in results. Risk of bias assessment will be used to consider the validity of results, not to determine eligibility.

### Data analysis and synthesis

Summary measures of intervention effect size with associated estimates of precision (95% CI) will be calculated for the outcomes (current status, QoL and well-being, confidence, efficacy and esteem) and endpoints (end of intervention, intervention follow-ups) of interest in each study. We anticipate that included studies will present current status data as categorical, which will be dichotomously coded as engaged or not engaged (see ‘Outcomes’ section) and psychological data as continuous using a range of different measures. The summary measure of intervention effect for current status will be odds ratio. The required statistics to calculate odds ratio will include intervention sample size total, post-intervention engaged sample size, control total and post-control engaged sample size. In accordance with the intention to treat (ITT) approach, participants lost to follow-up will be classified/analysed as non-engaged. Sensitivity analyses (for number of participants followed-up and number engaged in each group) will also be conducted.

The summary measure of intervention effect for psychological data will be the standardised mean difference or, more precisely, Hedges' (adjusted) *g*, which includes a correction term for small study bias. The required summary statistics will be size of sample, mean and standard deviation for each group for the outcomes and assessment points of interest. If these data are not reported, they will be calculated from other data reported in the paper or, if not possible, we will request data from first or corresponding author.

We will synthesise data, either qualitatively or quantitatively, for each outcome at each endpoint. The decision to synthesise data statistically will be taken by the review team after considering the importance of clinical and methodological variation among the studies and from visual inspection of forest plots. If data are pooled statistically, random effects models will be used to incorporate the assumption that true treatment effects vary among the included studies. Heterogeneity will be assessed by the *I*^2^ index, which describes the percentage of variability in effect estimates above that expected by chance, with values up to 40% considered unlikely to be important.

Potential sources of intervention effect size heterogeneity will be investigated in sub-groups stratified by pre-trial duration of current status, socioeconomic status, NEET target group status (e.g. offenders) and intervention components (e.g. type, frequency, duration, provider and setting). Sensitivity analyses will assess robustness of conclusions to variations in analytic approach, including separate syntheses that exclude studies with high risk of bias or shorter term follow-up. Publication bias will be examined using appropriate statistical techniques dependent on the type, quality and quantity of data available [33-35]. Risk of small study bias will be assessed by visual assessment of funnel symmetry in the plots of each trial's SMD against its standard error (SE) or by Egger's regression asymmetry test. To quality assure the review, it will be conducted in accordance with best practice guidance [36] and be reported using the PRISMA statement [37].

## Discussion

This systematic review will establish the current state of evidence concerning the effectiveness of interventions targeting young people not in education, employment or training. The number of youth classified as NEET is an increasing problem globally [1,5]. These disengaged youth are at risk of a range of adverse outcomes, such as reduced social and community participation, with resultant costs in terms of lost economic productivity and increased need for welfare, health and service provision as well as personal costs. The review will consider intervention strategies aimed at NEET populations targeting changes to employment status and indicators of psychological and social well-being. By examining a broad range of interventions (e.g. career development, educational, and psychological), and their implementation and delivery, this review will highlight gaps in current evidence base as well as examples of effective practice.

The current systematic review will focus on objective evidence of effectiveness for interventions by limiting the inclusion criteria pertaining to design, that is, only high-quality trials as critiqued against current best scientific practice [36] will be reviewed. Existing reviews on this population [23] have tended to adopt a traditional narrative perspective and, as such, are prone to selective citation, lack robust quality assessment of included evidence and examine heterogeneity in a descriptive manner. In order to maximise the potential evidence base, we will (i) examine all levels and types of intervention, (ii) review a range of NEET populations, irrespective of geographical or demographic situation (e.g. youth offenders) and (iii) pursue unpublished and grey literature held by local and national organisations working with this population (e.g. stakeholders and providers). By adopting this strategy, we will be less likely to exclude studies that undertook subgroup analyses by employment status and/or age but did not publish the findings in the abstract. We will contact the study authors for possible subgroup analyses and request any additional unpublished data. This will increase the comprehensiveness of the search strategy and therefore the quality of the final synthesis.

The quantity and quality of research included is, however, likely to be limited for a number of reasons. First, there are a broad range of providers and stakeholders working with NEET populations, including multiple local authority departments (e.g. housing, care, health etc.), as well as international, national and local aid organisations. The literature base reporting on interventions is therefore diverse, and useful information may be difficult to access (e.g. internal local authority project evaluations). Second, scientific rigour is a challenge in terms of controlling for confounds (multiple agencies interacting with the population at any given time) and identification of an appropriate control group. Third, given that interventions are frequently delivered by voluntary organisations, there is often limited funding for service evaluations, a desire to maximise access (at the potential expense of control groups) and a practical need to identify short-term success for self-sustainability. Programmes funded and/or delivered by either the voluntary or statutory sectors often do not comply with recognised quality thresholds for evidence, as a result of localised funding for relatively small-scale projects within a short-time scale of funding. Finally, the nature of the population makes long-term studies difficult, with potential cycles of temporary engagement followed by disengagement and difficulties in maintaining contact for collection of follow-up data.

A further area of potential limitation arises in the diversity of interventions that may emerge. There is likely to be substantial heterogeneity in terms of the type, structure and delivery of interventions. Whilst a non-restrictive approach to included interventions enables an audit of approaches, the variance may limit our ability to draw meaningful conclusions regarding differential effectiveness. Heterogeneity also has the potential to be problematic in terms of the outcomes measured.

To contextualise the findings, initial evidence will be presented at a network meeting with invited stakeholders including: voluntary sector representatives, statutory service commissioners and youth intervention teams. Following feedback on the contextualising of results, a report and executive summary will then be developed by the research team to aid translation of the findings into practice. The research will be disseminated at national and international conferences and submitted for peer-reviewed publication.

## Abbreviations

PRISMA: preferred reporting items for systematic reviews and meta-analyses; NEET: not in education, employment or training; WHO: World Health Organisation.

## Competing interests

The authors declare that they have no competing interests.

## Authors' contributions

EO is the co-principal investigator of this review and contributed to review conception, refinement of the design and writing the manuscript. LM is the co-principal investigator of this review and contributed to review conception, refinement of the design and writing the manuscript. CBr led the design of the protocol. HS assisted conception of the study, refinement of the design and provided population expertise. AO participated in the design of the review and protocol and contributed to drafting the manuscript. CBa participated in the design of the review and protocol and contributed to drafting the manuscript. CT participated in the design of the review and protocol and contributed to drafting the manuscript. All authors read and approved the final manuscript.
